# Development of a Rapid Tool for Metal Injection Molding Using Aluminum-Filled Epoxy Resins

**DOI:** 10.3390/polym15173513

**Published:** 2023-08-23

**Authors:** Chil-Chyuan Kuo, Xin-Yu Pan

**Affiliations:** 1 Department of Mechanical Engineering, Ming Chi University of Technology, No. 84, Gungjuan Road, New Taipei City 243, Taiwan; 2 Research Center for Intelligent Medical Devices, Ming Chi University of Technology, No. 84, Gungjuan Road, New Taipei City 243, Taiwan; 3 Department of Mechanical Engineering, Chang Gung University, No. 259, Wenhua 1st Road, Guishan District, Taoyuan City 333, Taiwan; 4 Center for Reliability Engineering, Ming Chi University of Technology, No. 84, Gungjuan Road, New Taipei City 243, Taiwan; 5 Shin Zu Shing Co., Ltd., No. 174, Junying Street, Shulin District, New Taipei City 238, Taiwan

**Keywords:** rapid tooling, metal injection molding, die life, manufacturing time, manufacturing cost

## Abstract

Metal injection molding (MIM) is a near net-shape manufacturing process combining conventional plastic injection molding and powder metallurgy. Two kinds of injections molds for MIM were developed using conventional mold steel and aluminum (Al)-filled epoxy resins in this study. The characteristics of the mold made by rapid tooling technology (RTT) were evaluated and compared with that of the fabricated conventional machining method through the MIM process. It was found that the service life of the injection mold fabricated by Al-filled epoxy resin is about 1300 molding cycles with the average surface roughness of 158 nm. The mold service life of the injection mold fabricated by Al-filled epoxy resin is about 1.3% that of the conventional mold steel. The reduction in manufacturing cost of an injection mold made by Al-filled epoxy resin is about 30.4% compared with that of the fabricated conventional mold steel. The saving in manufacturing time of an injection mold made by RTT is about 30.3% compared with that of the fabricated conventional machining method.

## 1. Introduction

Metal injection molding (MIM) is a near-net shape manufacturing approach for fabricating metal components with excellent mechanical properties [1,2]. In addition, the powder injection molding (PIM) offers a unique solution for the mass production of precision parts with excellent mechanical properties. The advantages of MIM involve high production rate, good mechanical properties, good dimensional control, and good shape complexity. The disadvantages include high sintering temperature, part size limitation, and product with residual pores. The powders used for MIM or PIM involve zirconia [3], 316L stainless steel [4,5], tungsten carbide [6,7], titanium [8,9], copper, chrome steel, and nickel. In general, the MIM process consists of four distinct steps, i.e., mixing, injection molding, de-binding, and sintering. Firstly, the metallic powders were mixed with binders to form a feedstock. The feedstock was then injected to cavity for manufacturing green parts using MIM machine. The binder of green parts can be removed by the de-binding process to form brown parts. Finally, brown parts were sintered at a high temperature and in a high-vacuum atmosphere to form final parts. Some studies regarding the MIM process have been carried out. Safarian et al. [10] investigated the effects of sintering parameters, such as dwell time, sintering temperature, and heating rate on diffusion bonding of 316L stainless steel in inserted metal injection molding. It was found that the sintering temperature is the most important parameter compared with the dwelling time and heating rate on the diffusion bonding of 316L stainless steel. Sahli et al. [11] developed a numerical model based on elastic–viscoplastic constitutive equations for calculating macroscopic deformation and structural evolution during the sintering of complex micro-gear compacts. Hayat et al. [12] developed a water-soluble PEG/PMMA binder system for μ-MIM. It was found that the developed binder systems are more suitable for an μ-MIM process that has an inherently higher cooling rate. Imgrund et al. [13] proposed two-component metal injection molding for the manufacturing of multi-functional micro-parts. It was found that the whole material interfaces of less than 500 µm × 500 µm can be obtained by the careful selection and tailoring of metal powders, injection molding and co-sintering parameters. Oh et al. [14] investigated the nano-powder effects on both the solvent and thermal de-binding processes. The results showed that the immersing time consuming in the solvent was increased by the nano-powder and the amount of the residual binder. Kate et al. [15] investigated the influence of feedstock properties on the injection molding of aluminum nitride. It was found that the estimated feedstock properties as input parameters in mold-filling simulations could be extended for a variety of material systems and geometries early in the design phase. Lamarre et al. [16] developed a new injection concept for increasing the moldability of powder–binder mixtures in the low-pressure powder injection molding. Zhang et al. [17] used three metallic powders with different compositions, particle shapes and particle sizes for producing microstructured parts using the injection molding process. The results showed that the microstructured parts can be obtained using the process chain of injection, de-binding and sintering with metallic powders. Zhang et al. [18] produced the M2 tool steel components with microstructures using the PIM process. Nayak et al. [19] developed powders to fabricate stainless steel parts using the PIM process. García et al. [20] fabricated steel matrix composites reinforced with different amounts of vanadium carbide (VC) using the MIM process and investigated the effects of adding VC on dry sliding wear behavior using both pin-on-disk and ball-on-flat tests. Dehghan-Manshadi et al. [21] reviewed recent developments in the MIM of titanium and its alloys as well as the outstanding challenges with a special focus on MIM of hydride–dehydride titanium powder. Thavanayagam et al. [22] investigated the effect of binder composition, powder loading, de-binding time and temperature on the de-binding rate for removing polyethylene glycol–polyvinyl butyral with water, and the porosity and microstructure of molded parts. Two materials have been proposed for fabricating mold inserts for MIM process, including silicone [23] and hardened steel [24].

The advantages of injection mold fabricated by conventional mold steel for MIM include machinability, cost-effectiveness, and thermal conductivity compared with injection mold fabricated by rapid tooling technology (RTT) [25,26,27,28,29,30]. However, these methods have disadvantages, including complicated manufacturing processes, long processing time, and high production cost. Inductively coupled plasma etching requires high production cost due to the complex manufacturing processes and long processing time. In addition, a conventional milling machine has limitations on the minimum size of the microstructures in a mold insert. RTT provides lower energy consumption and environmental impact as compared with conventional mold or die. To solve these drawbacks, a cost-effective method for fabricating an injection mold for MIM was demonstrated using RTT. In this study, injection molding simulation software Modlex3D was employed to determine the initial process parameter settings for MIM, the Al-filled epoxy resin were used to fabricate injection mold using RTT and applied to MIM. The characteristics of the injection mold fabricated by RTT were evaluated and compared with that fabricated conventional machining method through MIM process. The changes in the surface roughness of the mold surface after MIM were continuously recorded to evaluate the longevity of the fabricated injection molds. The difference between mold life, production cost and production time was investigated thoroughly.

## 2. Experimental Details

**Figure 1:**
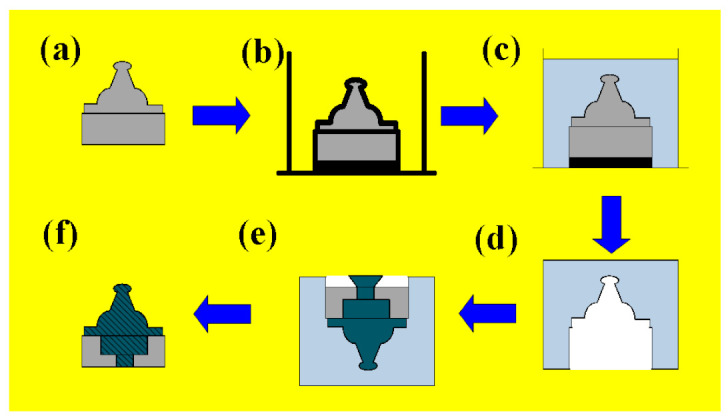
Detailed manufacturing process for fabricating a rapid tool for MIM: (**a**) Preparation of a master model, (**b**) placing the master model into the mold frame, (**c**) preparation of intermediary mold materials and casting, (**d**) completion of intermediate mold, (**e**) pouring Al-filled epoxy resins, and (**f**) finish of an injection mold for MIM.

A manufacturing process for fabricating an injection mold for the MIM process was developed. Figure 1 shows the detailed manufacturing process for fabricating a rapid tool for MIM. An intermediary mold which is complementary in shape to the injection mold was fabricated by both liquid silicone rubber (KE-1310ST, Shin Etsu Inc., New Taipei City, Taiwan). The Al-filled epoxy resin (TE 375, Jasdi Chemicals Inc., New Taipei City, Taiwan) was used to make an injection mold through the intermediary mold. A vacuum machine (F-600, Feiling) was used to eliminate air bubbles from the resulting mixture. The fabricated injection mold was then cured using a convection oven (DH400, Deng Yag Inc., New Taipei City, Taiwan) for obtaining the required mechanical properties. Finally, the fabricated injection mold was machined to the dimensions needed. To compare the performance of the injection molds fabricated by Al-filled epoxy resin, STAVAX-electro slag remelting (ESR) stainless mold steel (ASSAB Inc., New Taipei City, Taiwan) was also used to fabricate an injection mold. An injection machine (α-S100iA, FANUC Inc., Tokyo, Japan) was used to form green parts. In this study, a two-plate mold with a direct gate was used because of the simplest structure of the injection mold. In addition, two cavities in one mold base were used in this study because the performance of two different mold inserts can be evaluated under the same injection process parameters. Table 1 shows the MIM process parameters based on practical experience in the industry. The master model and injection mold were designed by using Pro/ENGINEER software. The dimensions of the master model were 15 mm in length, 15 mm in width, and 2 mm in thickness. The feedstock is the mixture of metal powder and binder for injection molding. The quality of feedstock is dependent on the type of metal powder and binder because the agglomeration, de-binding, particle packing, and dimensional correctness were affected by the type of binder employed. In this study, the metal matrix composite contains Fe and Ni powers. The polypropylene and paraffin wax of 50:50 ratio were mixed as a binder in this study. The metal powders (60 vol. %) and binder (40 vol. %) were warmed at 100 °C and then mixed in a mixer at 150 °C for 2 h. The average particle sizes of the metal powders were examined by field-emission scanning electron microscopy (SEM) (JEC3000-FC, JEOL Inc., Tokyo, Japan). The solvent (n-Heptane) de-binding process was used to remove the binder from the green parts. The de-binding time of solvent was about 6 h using a de-wax furnace (MIM-500D, Yu-He Inc.). The sintered products can be obtained from green parts using a vacuum-sintering furnace (VM-600, Mei-Yang Inc., New Taipei City, Taiwan). The sintering temperature was about 1300 °C with a heating rate of 5–10 °C/min under nitrogen purged atmosphere. The sintering time was about 24 h in the vacuum environment. In order to minimize the defects caused by the nature of the injection-molding process, Moldex 3D simulation software was used in this study because it provides the quality control tool for evaluating molding conditions. The service life of the two kinds of injection molds was carried out using an MIM injection machine. The value of center-line average surface roughness (Ra) was used to evaluate the changes in the surface roughness of the two kinds of injection molds. The measuring range is 250 µm × 250 µm. The WLI (7502, Chroma Inc., New Taipei City, Taiwan) was used to measure the surface roughness of the mold surface after MIM. The changes in surface roughness of the fabricated injection mold were investigated and compared with the injection mold fabricated by conventional mold steel. The micro-Vickers hardness of specimens was measured under the load of 2.9 N with 15 s using a micro-Vickers hardness tester (HM-112, Mitutoyo Akashi Inc., Taipei, Taiwan). Typical defects in the sintered parts such as porosity, cavities, inclusions and cracks were examined through an X-ray computed tomography (CT) scan (Tom tomoscope 200-190 3D CNC Werth Messtechnik GmbH Inc., Niedernhall, Germany).

## 3. Results and Discussion

**Figure 2:**
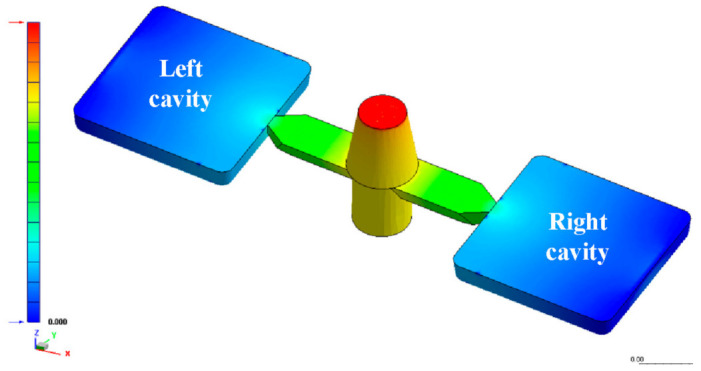
Schematic illustration of an injection mold with two cavities.

**Figure 3:**
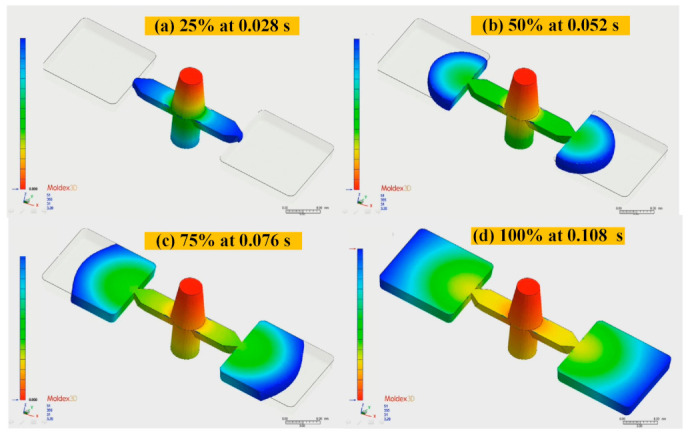
Simulation result of filling process.

**Figure 4:**
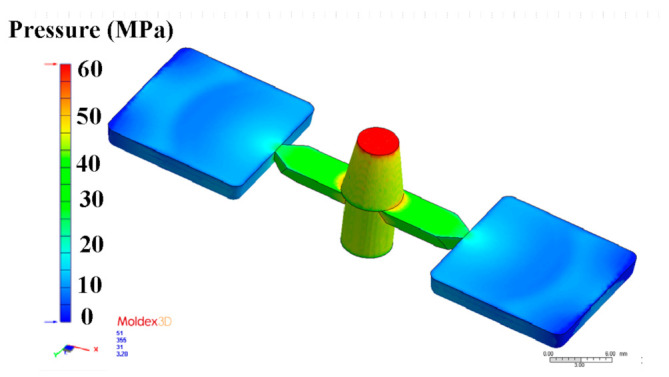
Simulation result of maximum injection pressure.

**Figure 5:**
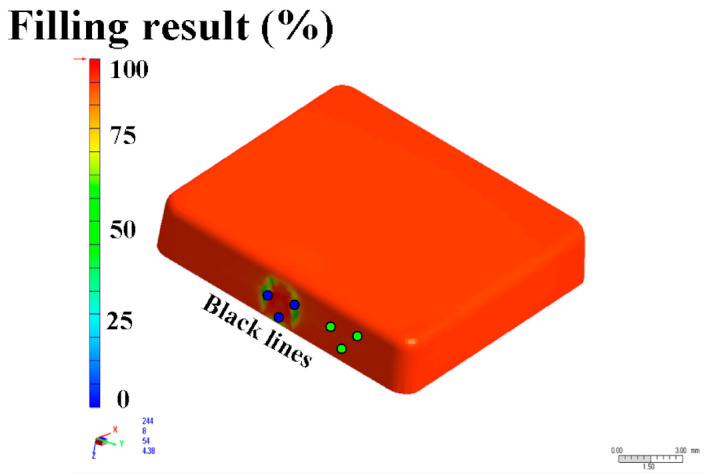
Simulation result of powder concentration distribution.

**Figure 6:**
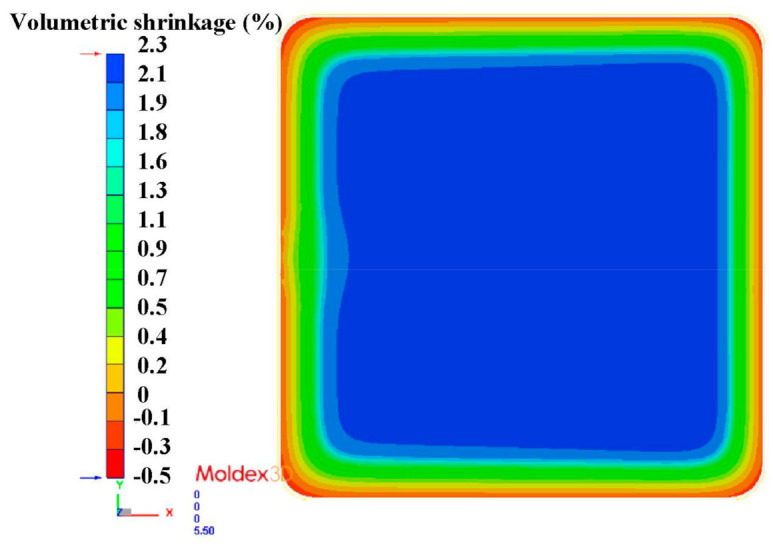
Simulation result of volume shrinkage.

**Figure 7:**
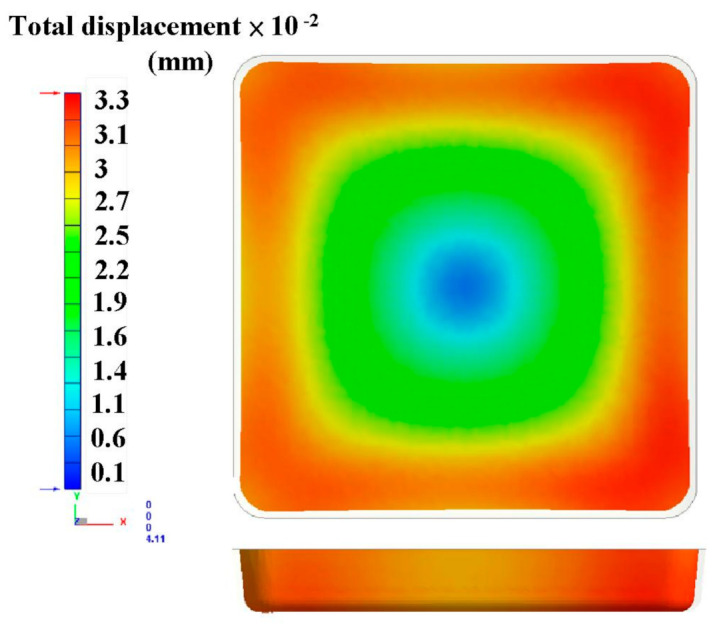
Simulation result of the warpage.

The metal injection-molding simulation software Moldex 3D can resolve and predict the metal injection-molding problems in the mold design stage. In this study, an injection mold with two cavities was designed for the MIM process, as shown in Figure 2. Figure 3 shows the simulation result of the filling process, and the filling time of the molded part is approximately 0.108 s. Figure 4 shows the simulation result of the maximum injection pressure. The maximum injection pressure is approximately 59.72 MPa. In general, the powder concentration distribution is usually unsteady. Figure 5 shows the simulation result of the powder concentration distribution. As can be seen, black-line marks were observed near the gate due to low powder concentration. Figure 6 shows the simulation result of volume shrinkage. The shrinkage of molded part is uniform, and the volume shrinkage is approximately 2.36% after injection molding [31]. Figure 7 shows the simulation result of the warpage. As a result, the molded part was deformed inward, and the total displacement of the warpage is approximately 0.034 mm.

**Figure 8:**
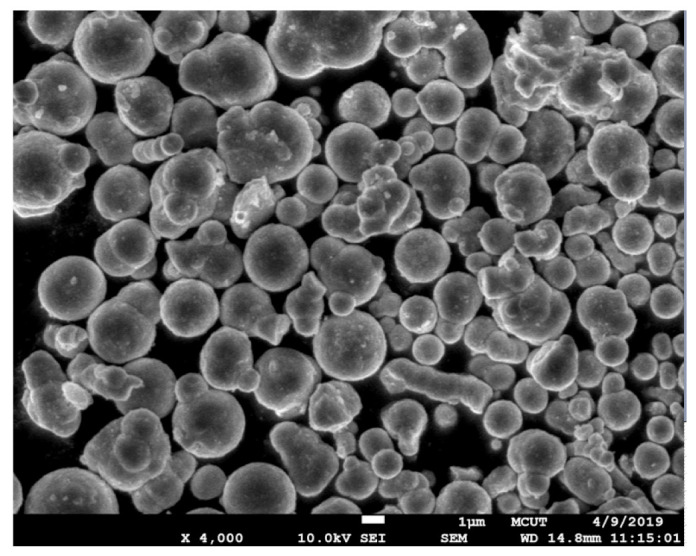
Morphology of the Fe powder.

**Figure 9:**
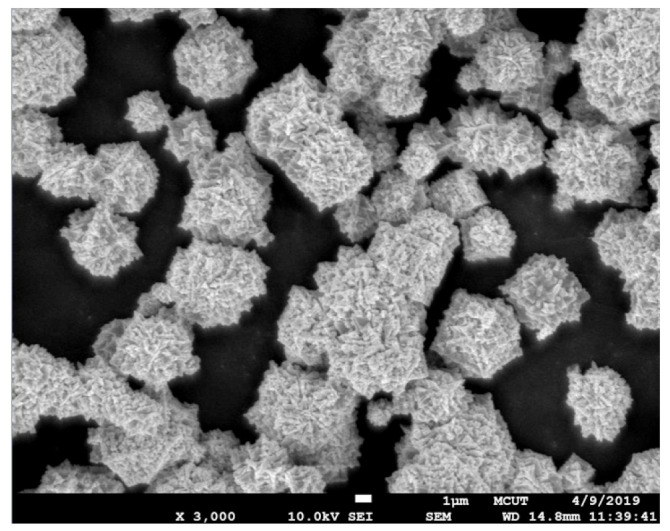
Morphology of the Ni powder.

**Figure 10:**
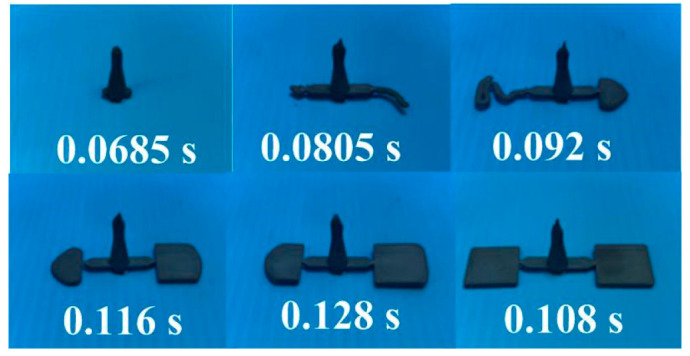
Results of the short shot test.

**Figure 11:**
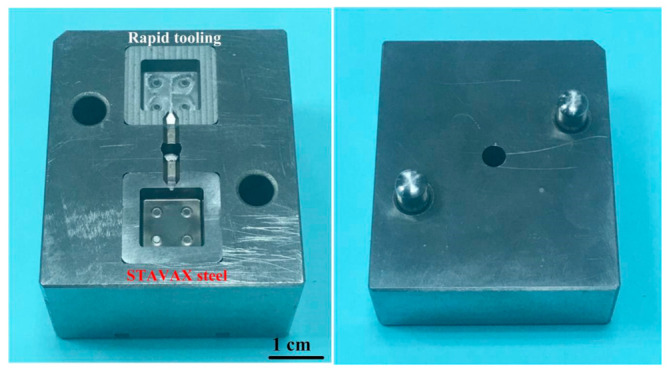
Injection molds fabricated by rapid tooling and STAVAX steel.

**Figure 12:**
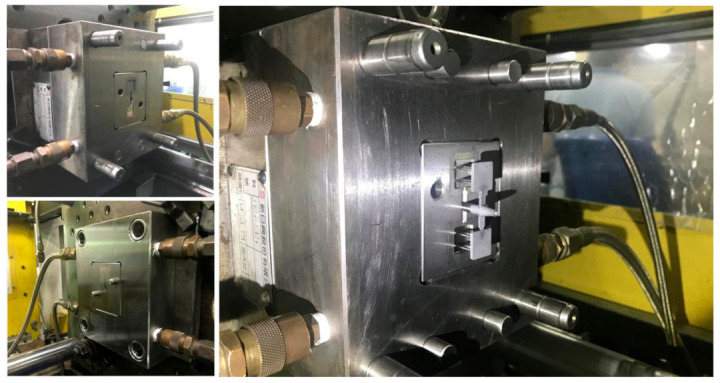
A green part was ejected during MIM process.

**Figure 13:**
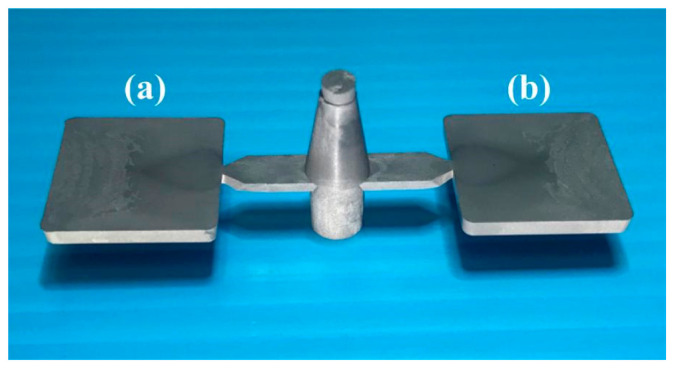
Green parts fabricated by (**a**) STAVAX steel and (**b**) Al-filled epoxy rapid tooling via the MIM process.

Figure 8 shows the morphology of the Fe powder. The average particle size of Fe powder is about 3 µm. Figure 9 shows the morphology of the Ni powder. The average particle size of Ni powder is about 6 µm. As can be seen, MIM powders have a spherical morphology. The average particle size can be estimated from these SEM micrographs. To evaluate the filling systems of the injection mold, the short shot test must be performed firstly; Figure 10 shows the results of the short shot test. As can be seen, the whole filling processes of the green part were similar to the simulation results [32]. Figure 11 shows the injection molds fabricated by rapid tooling and STAVAX steel. Figure 12 shows a green part was ejected during the MIM process. Figure 13 shows the green parts fabricated by STAVAX steel and Al-filled epoxy rapid tooling by the MIM process. The length, width and thickness of the molded part are 15 mm, 15 mm and 2 mm, respectively. In order to make the green parts easily release from the mold inserts, the draft angle of the molded part was designed as 5°. This result shows that the green part of Fe_2_Ni can be successfully fabricated by an injection mold fabricated by Al-filled epoxy rapid tooling.

**Figure 14:**
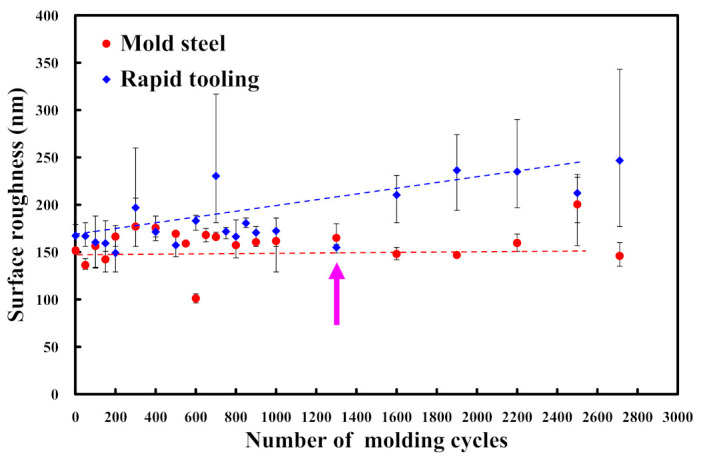
Average surface roughness of mold surface as a function of injection molding cycles.

**Figure 15:**
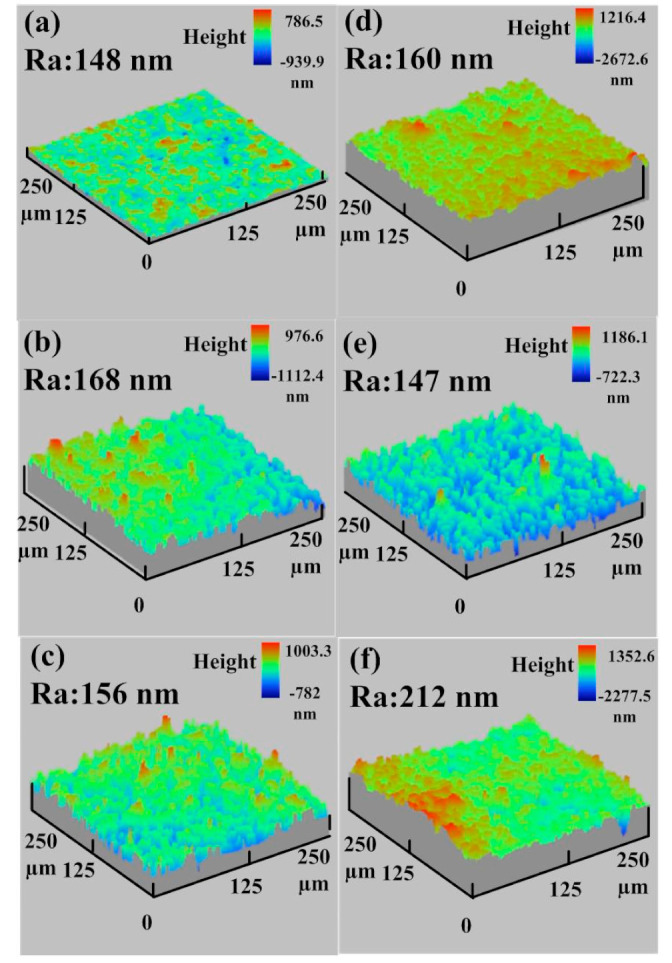
Average surface roughness of the injection mold fabricated by STAVAX stainless steel after injection molding cycles of (**a**) 1, (**b**) 500, (**c**) 900, (**d**) 1300, (**e**) 1600, and (**f**) 2500.

**Figure 16:**
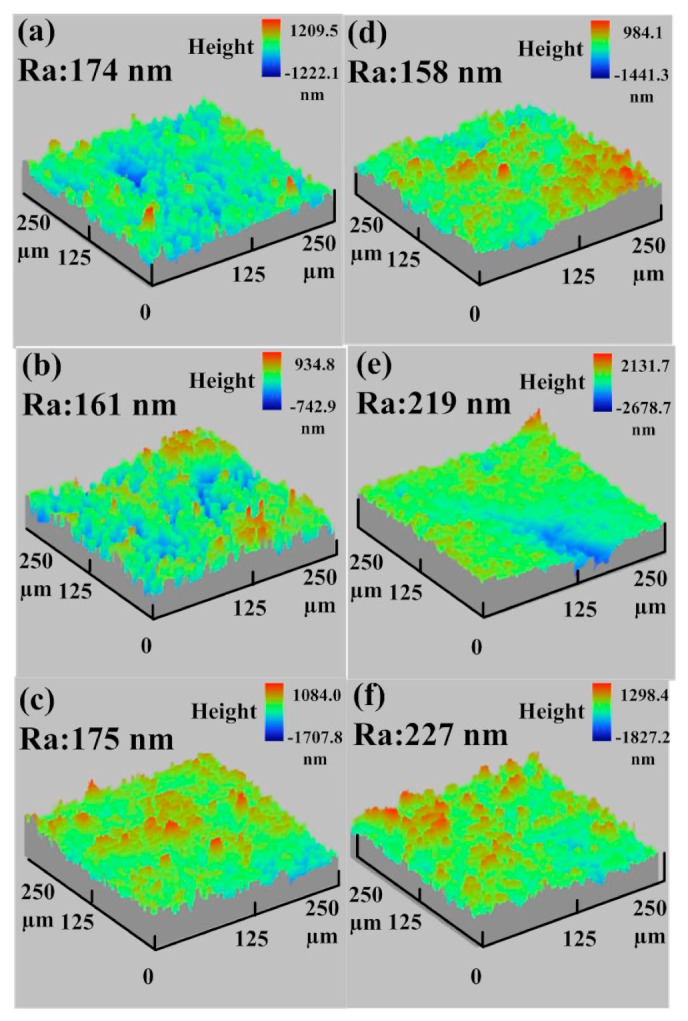
Average surface roughness of the injection mold fabricated by Al-filled epoxy resin after injection molding cycles of (**a**) 1, (**b**) 500, (**c**) 900, (**d**) 1300, (**e**) 1600, and (**f**) 2500.

In practice, the surface quality of the molded parts was affected by the surface roughness of the fabricated rapid tool. Thus, evaluating the changes in the surface roughness during the MIM process is crucial to assess the longevity of the manufactured rapid tool. To evaluate the longevity of injection molds made by the STAVAX stainless steel [33] and Al-filled epoxy resin, the MIM process was carried out with the feedstock of Fe_2_Ni. Figure 14 shows the average surface roughness of mold surface as a function of injection molding cycles. Figure 15 shows the average surface roughness of the injection mold fabricated by STAVAX stainless steel after 1 to 2500 injection molding cycles. As can be seen, the average surface roughness values of mold fabricated by STAVAX stainless steel after 1, 500, 900, 1300, 1600, 2500, and 2700 molding cycles were 148 nm, 168 nm, 156 nm, 160 nm, 147 nm, 212 nm, and 143 nm, respectively. Figure 16 shows the average surface roughness of the injection mold fabricated by Al-filled epoxy resin after 1 to 2500 injection molding cycles. The average surface roughness of the injection mold fabricated by Al-filled epoxy resin after 1, 500, 900, 1300, 1600, 2500, and 2700 molding cycles were 174 nm, 161 nm, 175 nm, 158 nm, 219 nm, 227 nm, and 220 nm, respectively. The results clearly show that the changes in the average surface roughness of the injection mold fabricated by Al-filled epoxy resin before 1300 molding cycles is very close to that of the injection mold fabricated by STAVAX stainless steel. However, the average surface roughness of the injection mold fabricated by Al-filled epoxy resin increased proportionally with increasing the injection mold cycles after 1300 molding cycles. This result showed that the service life of the injection mold fabricated by Al-filled epoxy resin is about 1300 molding cycles.

**Figure 17:**
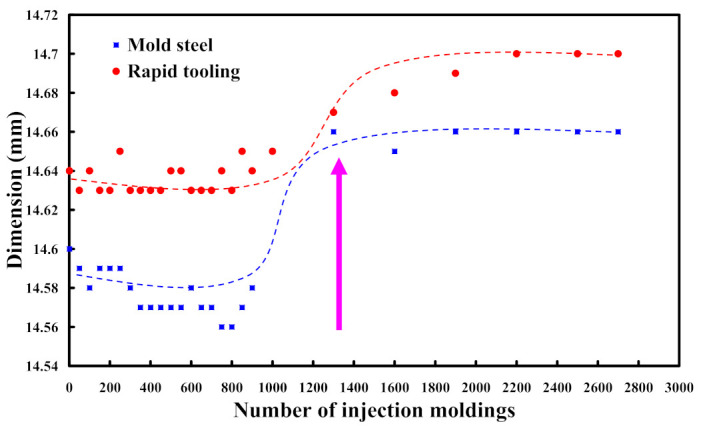
Dimension of the green part as a function of injection molding cycles.

To understand the dimensional accuracy of the green parts molded by mold steel and the injection molding tool, a series of experiments was performed [34]. Figure 17 shows the dimension of the green part as a function of injection molding cycles. As can be seen, the dimension of the green part increased rapidly after 1300 injection molding cycles, while the changes in the dimension of the green part were stabilized after injection molding cycles of 2000. The dimension of the green part fabricated by the mold made of STAVAX stainless steel after 1500, 900, 1300, 1600, 2500, and 2700 molding cycles was 14.6 mm, 14.57 mm, 14.58 mm, 14.66 mm, 14.65 mm, 14.66 mm, and 14.66 mm, respectively. The increase in the dimension of the green part was about 0.06 mm due to the wear of the mold surface after MIM process. The dimension of the green part fabricated by the mold made of Al-filled epoxy resin after 1500, 900, 1300, 1600, 2500, and 2700 molding cycles was 14.64 mm, 14.64 mm, 14.64 mm, 14.67 mm, 14.68 mm, 14.7 mm, and 14.7 mm, respectively. The increase in the dimension of the green part was also about 0.06 mm due to the wear on the mold surface after MIM process [35]. According to the changes in both dimension and surface roughness, it was found that the longevity of the injection mold fabricated by Al-filled epoxy resin is about 1300 molding cycles. In general, the longevity of the injection mold manufactured by conventional mold steel is 100,000 molding cycles. The mold service life of injection mold fabricated by Al-filled epoxy resin is about 1.3% of conventional mold steel.

**Figure 18:**
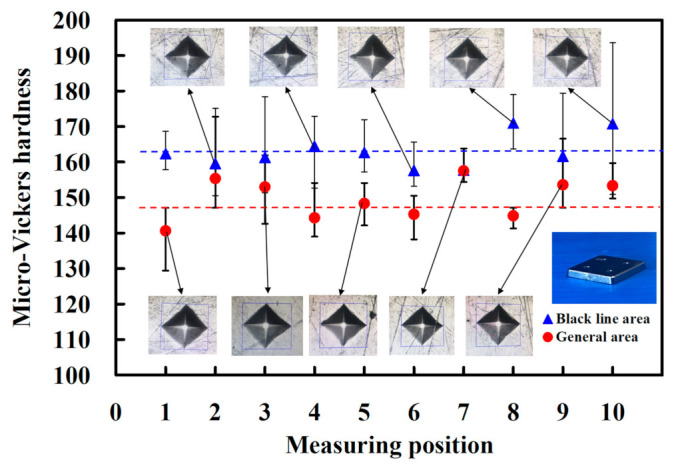
Micro-Vickers hardness of black-line area and general area in the sintered part.

To understand the difference in hardness [36] between the black-line area and general area in the sintered part, a series of experiments was performed. Figure 18 shows the micro-Vickers hardness of black-line area and general area in the sintered part. The average micro-Vickers hardness of the black-line area is about 162.9 HV, while the average micro-Vickers hardness of the general area is only about 149.6 HV. As a result, the average micro-Vickers hardness of the black-line is higher than those of the general area. This is because the metal powder near the sprue has a higher density than the general area due to the high-injection pressure during the MIM.

**Figure 19:**
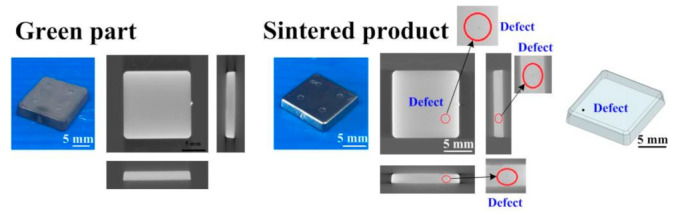
X-ray analysis of the green part and sintered product.

**Figure 20:**
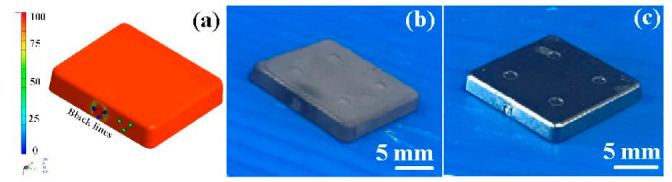
Black-line positions in (**a**) simulation result, (**b**) green part, and (**c**) sintered part.

**Figure 21:**
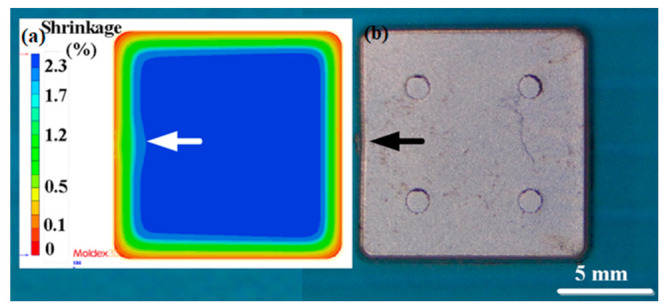
Volumetric shrinkage in (**a**) simulation result and (**b**) green part.

The final part made with Fe_2_Ni powders can be applied to the rotating shaft. The shrinkage of length, width, and thickness of the final part is about 22%. In order to evaluate the advantages of an injection mold fabricated by RT technology, both the production costs and manufacturing time of an injection mold fabricated by two different methods were investigated. Figure 19 shows the X-ray analysis of the green part and sintered product. The results clearly showed that no defects were found in the green part. However, some defects were found in the sintered products. This is because the defects that cannot be fully verified due to the polymer materials that exist in the green part will affect the inspection results. Therefore, the quality assurance operations of the MIM products still depend on the final sintered products. Figure 20 shows the black-line positions in the simulation result, green part, and sintered part. It should be noted that the black-line position in both the simulation result and green part is in the vicinity of the gate. In addition, the position of the black-line position predicted by simulation software is very close to the position of the green parts. This means that the Moldex 3D simulation solution can effectively predict the position of the black-line in the new MIM product development stage. Figure 21 shows the volumetric shrinkage in the simulation result and green part. It is interesting to note that there is obvious volumetric shrinkage in the vicinity of the gate. In addition, the volumetric shrinkage position predicted by simulation software is very close to the sintered part.

**Figure 22:**
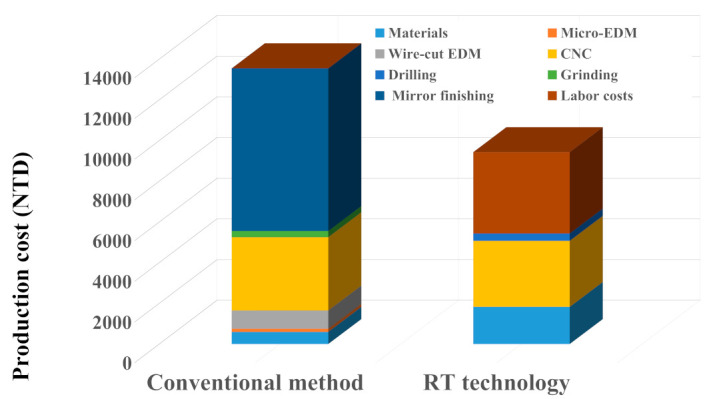
Production cost of the molds fabricated by conventional method and RT technology based on the valuation of the business department.

**Figure 23:**
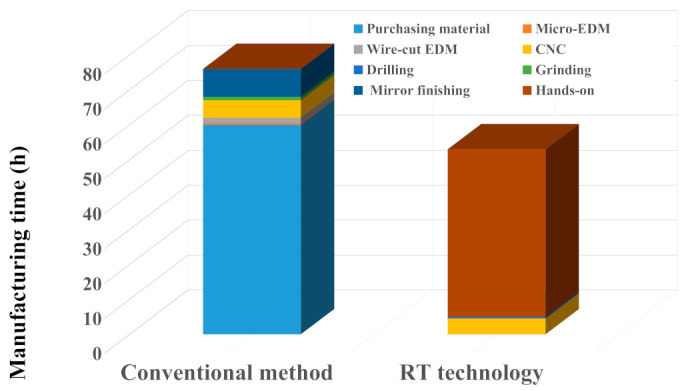
Manufacturing time of the molds fabricated by conventional method and RT technology based on the valuation of the business department.

Figure 22 shows the production cost of the molds fabricated by the conventional method and RT technology based on the valuation of the business department. The total production cost of an injection mold fabricated by the conventional method is about NT$ 13,550. The total production cost includes the mold material cost of NT$ 600, micro-hole machining cost of NT$ 150, wire-cut machining cost of NT$ 900, computer numerical control (CNC) machining cost of NT$ 3600, precision milling cost of NT$ 300, and mirror finish machining cost of NT$ 8000. The total production cost of an injection mold fabricated by RT technology is only about NT$ 9430. The total production cost involves the mold material cost of NT$ 1830, the labor cost of NT$ 4000, CNC machining cost of NT$ 3240, and precision drilling cost of NT$ 360. Thus, the saving in the production cost of an injection mold made by Al-filled epoxy resin is about 30.4% compared with that of the fabricated conventional mold steel. Figure 23 shows the manufacturing time of the molds fabricated by the conventional method and RT technology based on the valuation of the business department. The total manufacturing time of an injection mold fabricated by the conventional method is about 76 h. The total manufacturing time includes the mold material preparation time of 60 h, micro-hole machining time of 0.5 h, wire-cut machining time of 1.5 h, CNC machining time of 5 h, precision milling time of 1 h, and mirror finish machining time of 8 h. The total manufacturing time of an injection mold fabricated by RT technology is only 53 h. The total manufacturing time includes the mold manufacturing time of 48 h, CNC machining time [37,38] of 4.5 h, and precision drilling time [39] of 0.5. Thus, the saving in the manufacturing time of an injection mold made by RT technology is about 30.3% compared with that of the fabricated conventional machining method. These results obtained are very practical and economical for making large-sized injection molds [40] for the MIM process and offer potential for many applications in the MIM industry. Especially, the results obtained in this work also meet the sustainable development goal 12 due to lower environmental impact as compared with the injection mold or die obtained by conventional machining approaches. However, the inherent limitations of an injection molding tool fabricated with Al-filled epoxy resin include their low mechanical properties and heat transfer capability compared with STAVAX stainless steel [41]. These limitations can further be improved by adding copper powders [42,43], molybdenum disulfide [44,45,46,47], zirconia ceramics [48,49], or silicon nitride ceramics [50,51,52,53] particles in the mixture. These issues are currently being investigated, and the results will be presented in a later study.

## 4. Conclusions

MIM is a near-net shaping approach for producing metallic parts with high intricate shape and good mechanical properties. The main objective of this work is to develop a cost-effective method for fabricating an injection mold for MIM. A new metal part can be fabricated by an injection mold fabricated by conventional mold steel. However, it is not an effective way for a new metallic part in the research and development stages due to high production cost and high risk. The metallic components with high density can be fabricated by the MIM process integrating powder metallurgy and plastic injection molding. The main conclusions from the experimental work in this study are as follows:

1. The findings of this study are very practical and provide the greatest application potential in the research and development stage of a new metal part.
2. The reduction in the manufacturing time of an injection mold made by RT technology is about 30.3% compared with that of the fabricated conventional machining method.
3. The reduction in production cost of an injection mold by Al-filled epoxy resin is about 30.4% compared with that of the fabricated conventional mold steel.
4. The longevity of the injection mold fabricated by Al-filled epoxy resin is about 1300 molding cycles. The mold service life of an injection mold fabricated by Al-filled epoxy resin is about 1.3% of that of conventional mold steel.
5. The copper powder, molybdenum disulfide, zirconia ceramics, or silicon nitride ceramics particles were recommended to add to the mixture to improve the mechanical properties and heat transfer capability of Al-filled epoxy resin.

## Figures and Tables

**Table 1:** MIM process parameters based on practical experience in the industry.

Parameters	Value
Injection time (s)	0.108
Injection pressure (MPa)	70
Injection speed (mm/s)	80
Packing pressure (MPa)	50
Packing time (s)	1
Molding temperature (°C)	160
Mold temperature (°C)	20

## Data Availability

The data presented in this study are available on request from the corresponding author.
